# Bis(2-methyl-1*H*-benzimidazol-3-ium) naphthalene-1,5-disulfonate

**DOI:** 10.1107/S1600536812039396

**Published:** 2012-09-22

**Authors:** Shuai-Shuai Wei, Shou-Wen Jin, Qiong Dong, Xin-Chao Cao, Ze-Yun Yu

**Affiliations:** a Tianmu College of ZheJiang A & F University, Lin’An 311300, People’s Republic of China; b Faculty of Science, ZheJiang A & F University, Lin’An 311300, People’s Republic of China

## Abstract

The asymmetric unit of the title compound, 2C_8_H_9_N_2_^+^·C_10_H_6_O_6_S_2_^2−^, contains a 2-methyl­benzimidazolium cation and one half of a naphthalene-1,5-disulfonate anion. The formula unit is generated by an inversion center. In the crystal, N—H⋯O hydrogen bonds link the components into chains along [001]. In addition, weak C—H⋯O hydrogen bonds and weak C—H⋯π inter­actions are observed. The methyl H atoms were refined as disordered over two sets of sites with equal occupancy.

## Related literature

For general background to organic acids, see: Jin *et al.* (2012 ▶); Elder *et al.* (2010 ▶); Voogt & Blanch (2005 ▶); Wang *et al.* (2005 ▶); Zhang *et al.* (2005 ▶).
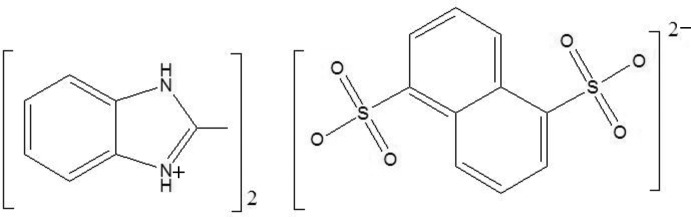


## Experimental

### 

#### Crystal data

2C_8_H_9_N_2_^+^·C_10_H_6_O_6_S_2_^2−^
*M* *_r_* = 552.61
Triclinic, 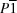

*a* = 8.0360 (7) Å
*b* = 9.3969 (8) Å
*c* = 9.5101 (9) Å
α = 105.789 (1)°
β = 103.303 (1)°
γ = 106.497 (2)°
*V* = 624.75 (10) Å^3^
*Z* = 1
Mo *K*α radiation
μ = 0.26 mm^−1^
*T* = 298 K
0.45 × 0.41 × 0.19 mm

#### Data collection

Bruker SMART CCD diffractometer
Absorption correction: multi-scan (*SADABS*; Bruker, 2002 ▶) *T*_min_ = 0.888, *T*_max_ = 0.951
3137 measured reflections
2169 independent reflections
1694 reflections with *I* > 2σ(*I*)
*R*_int_ = 0.030

#### Refinement

*R*[*F* ^2^ > 2σ(*F* ^2^)] = 0.043
*wR*(*F* ^2^) = 0.119
*S* = 1.05
2169 reflections
173 parameters
H-atom parameters constrained
Δρ_max_ = 0.28 e Å^−3^
Δρ_min_ = −0.37 e Å^−3^

### 

Data collection: *SMART* (Bruker, 2002 ▶); cell refinement: *SAINT* (Bruker, 2002 ▶); data reduction: *SAINT*; program(s) used to solve structure: *SHELXS97* (Sheldrick, 2008 ▶); program(s) used to refine structure: *SHELXL97* (Sheldrick, 2008 ▶); molecular graphics: *PLATON* (Spek, 2009 ▶); software used to prepare material for publication: *SHELXTL* (Sheldrick, 2008 ▶).

## Supplementary Material

Crystal structure: contains datablock(s) global, I. DOI: 10.1107/S1600536812039396/lh5531sup1.cif

Structure factors: contains datablock(s) I. DOI: 10.1107/S1600536812039396/lh5531Isup2.hkl

Supplementary material file. DOI: 10.1107/S1600536812039396/lh5531Isup3.cml

Additional supplementary materials:  crystallographic information; 3D view; checkCIF report

## Figures and Tables

**Table 1:** Hydrogen-bond geometry (Å, °) *Cg*1 and *Cg*2 are the centroids of the C9–C11/C11^i^/C12^i^/C13^i^ and C11–C13/C9^i^/C10^i^/C11^i^ rings, respectively [symmetry code: (i) −*x*, −*y* + 1, −*z* + 1].

*D*—H⋯*A*	*D*—H	H⋯*A*	*D*⋯*A*	*D*—H⋯*A*
N1—H1⋯O2^ii^	0.86	1.86	2.704 (3)	165
N2—H2⋯O1	0.86	1.88	2.684 (3)	155
C8—H8*E*⋯O3^iii^	0.96	2.32	3.230 (4)	158
C4—H4⋯*Cg*1^iv^	0.93	2.61	3.468 (3)	154
C4—H4⋯*Cg*2^v^	0.93	2.61	3.468 (3)	154

Symmetry codes: (ii) 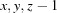; (iii) 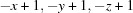; (iv) 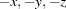; (v) 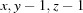.

**Fig. 1.**
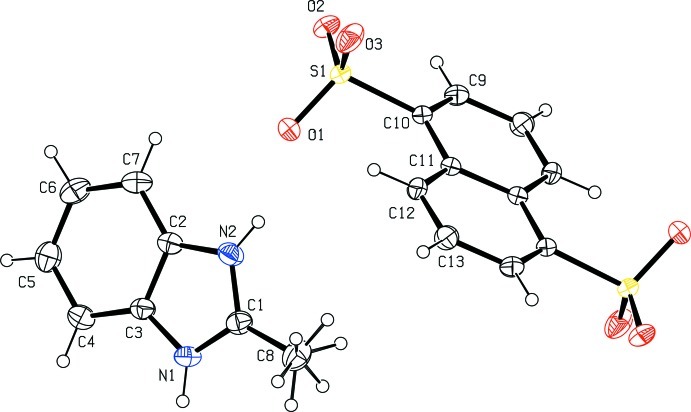
The molecular structure of the title compound with displacement ellipsoids drawn at the 30% probability level. Only the symmetry unique cation is shown and in the anion unlabeled atoms are related by the symmetry operator (-x, -y + 1, -z + 1).

**Fig. 2.**
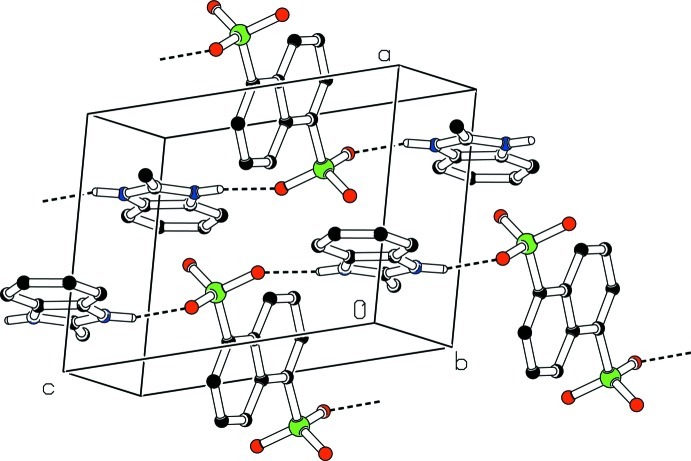
Part of the crystal structure with hydrogen bonds shown as dashed lines. H atoms not involved in the hydrogen bonds have been omitted for clarity.

## supplementary crystallographic information

### Comment

Sulfonic acids are important compounds, which have been widely used in various fields as coordination chemistry (Wang *et al.*, 2005), pharmaceutical chemistry (Elder *et al.*, 2010), and supramolecular chemistry (Voogt & Blanch, 2005). Recently the main focus for sulfonic acids has been in crystal engineering *via* hydrogen bonded assembly of sulfonic acid and organic base (Zhang *et al.*, 2005). As an extension of our study concentrating on hydrogen bonded assemblies of organic acids and organic bases (Jin *et al.*, 2012) herein we report the crystal structure of the title compound (I).

The molecular structure of (I) is shown in Fig. 1. The anion lies across an inversion center. In the crystal, N—H···O hydrogen bonds link the components into chains along [001] (Fig. 2). In addition, weak C—H···O hydrogen bonds and weak C—H···π interactions are observed.

### Experimental

2-Methyl-1*H*-benzimidazole (24.0 mg, 0.20 mmol) was dissolved in 10 ml of methanol, and naphthalene-1,5-disulfonic acid tetrahydrate (36 mg, 0.1 mmol) was added. The solution was stirred for 1 h, and then filtered into a test tube. The solution was left standing at room temperature for about one week whereupon colorless block crystals were obtained.

### Refinement

All H atoms were positioned geometrically with C—H = 0.93–0.96 Å, N—H = 0.86 Å and constrained to ride on their parent atoms with *U*_iso_(H) = 1.2*U*_eq_(C,*N*). The methyl H atoms were refined as disordered over six sites with equal occupancy.

### Crystal data

**Table:**

2C_8_H_9_N_2_^+^·C_10_H_6_O_6_S_2_^2^^−^	*Z* = 1
*M**_r_* = 552.61	*F*(000) = 288
Triclinic, *P*1	*D*_x_ = 1.469 Mg m^−^^3^
Hall symbol: -P 1	Mo *K*α radiation, λ = 0.71073 Å
*a* = 8.0360 (7) Å	Cell parameters from 1471 reflections
*b* = 9.3969 (8) Å	θ = 2.4–28.0°
*c* = 9.5101 (9) Å	µ = 0.26 mm^−^^1^
α = 105.789 (1)°	*T* = 298 K
β = 103.303 (1)°	Block, colourless
γ = 106.497 (2)°	0.45 × 0.41 × 0.19 mm
*V* = 624.75 (10) Å^3^	

### Data collection

**Table:**

Bruker SMART CCD diffractometer	2169 independent reflections
Radiation source: fine-focus sealed tube	1694 reflections with *I* > 2σ(*I*)
Graphite monochromator	*R*_int_ = 0.030
φ and ω scans	θ_max_ = 25.0°, θ_min_ = 2.4°
Absorption correction: multi-scan (*SADABS*; Bruker, 2002)	*h* = −9→9
*T*_min_ = 0.888, *T*_max_ = 0.951	*k* = −8→11
3137 measured reflections	*l* = −10→11

### Refinement

**Table:**

Refinement on *F*^2^	Secondary atom site location: difference Fourier map
Least-squares matrix: full	Hydrogen site location: inferred from neighbouring sites
*R*[*F*^2^ > 2σ(*F*^2^)] = 0.043	H-atom parameters constrained
*wR*(*F*^2^) = 0.119	*w* = 1/[σ^2^(*F*_o_^2^) + (0.0583*P*)^2^ + 0.1477*P*] where *P* = (*F*_o_^2^ + 2*F*_c_^2^)/3
*S* = 1.05	(Δ/σ)_max_ < 0.001
2169 reflections	Δρ_max_ = 0.28 e Å^−^^3^
173 parameters	Δρ_min_ = −0.37 e Å^−^^3^
0 restraints	Extinction correction: *SHELXL97* (Sheldrick, 2008), Fc^*^=kFc[1+0.001xFc^2^λ^3^/sin(2θ)]^-1/4^
Primary atom site location: structure-invariant direct methods	Extinction coefficient: 0.043 (6)

### Special details

**Table:**

Geometry. All e.s.d.'s (except the e.s.d. in the dihedral angle between two l.s. planes) are estimated using the full covariance matrix. The cell e.s.d.'s are taken into account individually in the estimation of e.s.d.'s in distances, angles and torsion angles; correlations between e.s.d.'s in cell parameters are only used when they are defined by crystal symmetry. An approximate (isotropic) treatment of cell e.s.d.'s is used for estimating e.s.d.'s involving l.s. planes.
Refinement. Refinement of *F*^2^ against ALL reflections. The weighted *R*-factor *wR* and goodness of fit *S* are based on *F*^2^, conventional *R*-factors *R* are based on *F*, with *F* set to zero for negative *F*^2^. The threshold expression of *F*^2^ > σ(*F*^2^) is used only for calculating *R*-factors(gt) *etc*. and is not relevant to the choice of reflections for refinement. *R*-factors based on *F*^2^ are statistically about twice as large as those based on *F*, and *R*-factors based on ALL data will be even larger.

### Fractional atomic coordinates and isotropic or equivalent isotropic displacement parameters (Å^2^)

**Table:**

	*x*	*y*	*z*	*U*_iso_*/*U*_eq_	Occ. (<1)
N1	0.2089 (3)	0.0991 (2)	−0.0901 (2)	0.0390 (5)	
H1	0.1838	0.1027	−0.1817	0.047*	
N2	0.2562 (3)	0.1632 (2)	0.1561 (2)	0.0404 (5)	
H2	0.2664	0.2148	0.2495	0.048*	
O1	0.3057 (3)	0.2484 (2)	0.4604 (2)	0.0530 (6)	
O2	0.1946 (3)	0.1418 (2)	0.6381 (2)	0.0447 (5)	
O3	0.4010 (3)	0.4162 (2)	0.7288 (2)	0.0524 (5)	
S1	0.25771 (8)	0.28337 (7)	0.60043 (6)	0.0330 (2)	
C1	0.2166 (4)	0.2092 (3)	0.0356 (3)	0.0383 (6)	
C2	0.2784 (3)	0.0185 (3)	0.1067 (3)	0.0354 (6)	
C3	0.2475 (3)	−0.0230 (3)	−0.0511 (3)	0.0354 (6)	
C4	0.2607 (4)	−0.1614 (3)	−0.1376 (3)	0.0436 (7)	
H4	0.2379	−0.1902	−0.2438	0.052*	
C5	0.3095 (4)	−0.2543 (3)	−0.0584 (3)	0.0495 (7)	
H5	0.3195	−0.3484	−0.1125	0.059*	
C6	0.3443 (4)	−0.2105 (3)	0.1010 (3)	0.0497 (7)	
H6	0.3796	−0.2751	0.1506	0.060*	
C7	0.3281 (4)	−0.0747 (3)	0.1870 (3)	0.0455 (7)	
H7	0.3493	−0.0467	0.2929	0.055*	
C8	0.1861 (4)	0.3582 (3)	0.0410 (4)	0.0552 (8)	
H8A	0.1993	0.4176	0.1452	0.083*	0.50
H8B	0.0643	0.3332	−0.0263	0.083*	0.50
H8C	0.2749	0.4206	0.0077	0.083*	0.50
H8D	0.1597	0.3633	−0.0608	0.083*	0.50
H8E	0.2947	0.4477	0.1106	0.083*	0.50
H8F	0.0841	0.3603	0.0767	0.083*	0.50
C9	−0.0943 (3)	0.2568 (3)	0.5733 (3)	0.0356 (6)	
H9	−0.1013	0.1681	0.6009	0.043*	
C10	0.0652 (3)	0.3429 (3)	0.5591 (2)	0.0284 (5)	
C11	0.0793 (3)	0.4782 (3)	0.5137 (2)	0.0275 (5)	
C12	0.2405 (3)	0.5702 (3)	0.4963 (3)	0.0336 (6)	
H12	0.3438	0.5427	0.5143	0.040*	
C13	0.2477 (3)	0.6986 (3)	0.4535 (3)	0.0388 (6)	
H13	0.3552	0.7570	0.4421	0.047*	

### Atomic displacement parameters (Å^2^)

**Table:**

	*U*^11^	*U*^22^	*U*^33^	*U*^12^	*U*^13^	*U*^23^
N1	0.0449 (13)	0.0439 (13)	0.0288 (11)	0.0159 (11)	0.0095 (10)	0.0171 (10)
N2	0.0476 (14)	0.0419 (13)	0.0294 (11)	0.0160 (11)	0.0130 (10)	0.0101 (10)
O1	0.0700 (14)	0.0829 (15)	0.0407 (11)	0.0547 (12)	0.0338 (10)	0.0335 (10)
O2	0.0573 (12)	0.0481 (11)	0.0447 (10)	0.0312 (10)	0.0162 (9)	0.0290 (9)
O3	0.0407 (11)	0.0512 (12)	0.0517 (12)	0.0217 (10)	−0.0051 (9)	0.0104 (9)
S1	0.0378 (4)	0.0441 (4)	0.0266 (3)	0.0251 (3)	0.0108 (3)	0.0166 (3)
C1	0.0355 (14)	0.0395 (15)	0.0375 (14)	0.0113 (12)	0.0089 (11)	0.0155 (12)
C2	0.0355 (14)	0.0385 (14)	0.0301 (13)	0.0099 (11)	0.0107 (11)	0.0136 (11)
C3	0.0368 (14)	0.0395 (14)	0.0319 (13)	0.0130 (12)	0.0118 (11)	0.0166 (11)
C4	0.0476 (17)	0.0448 (16)	0.0327 (13)	0.0148 (13)	0.0114 (12)	0.0095 (12)
C5	0.0500 (17)	0.0393 (16)	0.0580 (18)	0.0182 (14)	0.0170 (15)	0.0149 (14)
C6	0.0512 (18)	0.0452 (17)	0.0570 (18)	0.0174 (14)	0.0127 (14)	0.0298 (15)
C7	0.0518 (17)	0.0488 (17)	0.0369 (14)	0.0144 (14)	0.0125 (13)	0.0235 (13)
C8	0.0558 (19)	0.0448 (17)	0.0609 (19)	0.0220 (15)	0.0107 (16)	0.0169 (15)
C9	0.0424 (15)	0.0397 (14)	0.0350 (13)	0.0185 (12)	0.0167 (12)	0.0221 (12)
C10	0.0315 (13)	0.0366 (13)	0.0225 (11)	0.0181 (11)	0.0092 (10)	0.0126 (10)
C11	0.0318 (13)	0.0336 (13)	0.0214 (11)	0.0162 (11)	0.0100 (10)	0.0112 (10)
C12	0.0286 (13)	0.0467 (15)	0.0353 (13)	0.0211 (12)	0.0137 (11)	0.0194 (12)
C13	0.0329 (14)	0.0477 (15)	0.0463 (15)	0.0160 (12)	0.0194 (12)	0.0261 (13)

### Geometric parameters (Å, º)

**Table:**

N1—C1	1.327 (3)	C6—H6	0.9300
N1—C3	1.390 (3)	C7—H7	0.9300
N1—H1	0.8600	C8—H8A	0.9600
N2—C1	1.335 (3)	C8—H8B	0.9600
N2—C2	1.389 (3)	C8—H8C	0.9600
N2—H2	0.8600	C8—H8D	0.9600
O1—S1	1.4491 (17)	C8—H8E	0.9600
O2—S1	1.4543 (17)	C8—H8F	0.9600
O3—S1	1.4427 (19)	C9—C10	1.366 (3)
S1—C10	1.786 (2)	C9—C13^i^	1.403 (4)
C1—C8	1.478 (4)	C9—H9	0.9300
C2—C3	1.386 (3)	C10—C11	1.433 (3)
C2—C7	1.390 (4)	C11—C12	1.413 (3)
C3—C4	1.384 (3)	C11—C11^i^	1.436 (4)
C4—C5	1.378 (4)	C12—C13	1.365 (3)
C4—H4	0.9300	C12—H12	0.9300
C5—C6	1.395 (4)	C13—C9^i^	1.403 (4)
C5—H5	0.9300	C13—H13	0.9300
C6—C7	1.375 (4)		
			
C1—N1—C3	109.56 (19)	H8A—C8—H8B	109.5
C1—N1—H1	125.2	C1—C8—H8C	109.5
C3—N1—H1	125.2	H8A—C8—H8C	109.5
C1—N2—C2	109.1 (2)	H8B—C8—H8C	109.5
C1—N2—H2	125.5	C1—C8—H8D	109.5
C2—N2—H2	125.5	H8A—C8—H8D	141.1
O3—S1—O1	112.92 (13)	H8B—C8—H8D	56.3
O3—S1—O2	113.16 (11)	H8C—C8—H8D	56.3
O1—S1—O2	111.54 (11)	C1—C8—H8E	109.5
O3—S1—C10	106.00 (11)	H8A—C8—H8E	56.3
O1—S1—C10	106.50 (10)	H8B—C8—H8E	141.1
O2—S1—C10	106.07 (11)	H8C—C8—H8E	56.3
N1—C1—N2	108.8 (2)	H8D—C8—H8E	109.5
N1—C1—C8	125.5 (2)	C1—C8—H8F	109.5
N2—C1—C8	125.7 (2)	H8A—C8—H8F	56.3
C3—C2—N2	106.4 (2)	H8B—C8—H8F	56.3
C3—C2—C7	121.8 (2)	H8C—C8—H8F	141.1
N2—C2—C7	131.7 (2)	H8D—C8—H8F	109.5
C4—C3—C2	121.7 (2)	H8E—C8—H8F	109.5
C4—C3—N1	132.2 (2)	C10—C9—C13^i^	120.3 (2)
C2—C3—N1	106.1 (2)	C10—C9—H9	119.8
C5—C4—C3	116.6 (2)	C13^i^—C9—H9	119.8
C5—C4—H4	121.7	C9—C10—C11	121.2 (2)
C3—C4—H4	121.7	C9—C10—S1	118.13 (18)
C4—C5—C6	121.5 (3)	C11—C10—S1	120.63 (17)
C4—C5—H5	119.2	C12—C11—C10	123.4 (2)
C6—C5—H5	119.2	C12—C11—C11^i^	118.9 (2)
C7—C6—C5	122.0 (3)	C10—C11—C11^i^	117.7 (3)
C7—C6—H6	119.0	C13—C12—C11	121.3 (2)
C5—C6—H6	119.0	C13—C12—H12	119.4
C6—C7—C2	116.2 (2)	C11—C12—H12	119.4
C6—C7—H7	121.9	C12—C13—C9^i^	120.5 (2)
C2—C7—H7	121.9	C12—C13—H13	119.7
C1—C8—H8A	109.5	C9^i^—C13—H13	119.7
C1—C8—H8B	109.5		
			
C3—N1—C1—N2	−1.0 (3)	C3—C2—C7—C6	−0.2 (4)
C3—N1—C1—C8	179.1 (3)	N2—C2—C7—C6	−177.4 (3)
C2—N2—C1—N1	1.1 (3)	C13^i^—C9—C10—C11	−1.3 (4)
C2—N2—C1—C8	−178.9 (3)	C13^i^—C9—C10—S1	178.19 (18)
C1—N2—C2—C3	−0.9 (3)	O3—S1—C10—C9	−118.6 (2)
C1—N2—C2—C7	176.6 (3)	O1—S1—C10—C9	120.9 (2)
N2—C2—C3—C4	179.3 (2)	O2—S1—C10—C9	2.0 (2)
C7—C2—C3—C4	1.5 (4)	O3—S1—C10—C11	60.9 (2)
N2—C2—C3—N1	0.3 (3)	O1—S1—C10—C11	−59.6 (2)
C7—C2—C3—N1	−177.5 (2)	O2—S1—C10—C11	−178.54 (17)
C1—N1—C3—C4	−178.4 (3)	C9—C10—C11—C12	−179.5 (2)
C1—N1—C3—C2	0.4 (3)	S1—C10—C11—C12	1.1 (3)
C2—C3—C4—C5	−1.2 (4)	C9—C10—C11—C11^i^	1.1 (4)
N1—C3—C4—C5	177.5 (3)	S1—C10—C11—C11^i^	−178.4 (2)
C3—C4—C5—C6	−0.2 (4)	C10—C11—C12—C13	−179.7 (2)
C4—C5—C6—C7	1.4 (5)	C11^i^—C11—C12—C13	−0.2 (4)
C5—C6—C7—C2	−1.2 (4)	C11—C12—C13—C9^i^	0.4 (4)

Symmetry code: (i) −*x*, −*y*+1, −*z*+1.

### Hydrogen-bond geometry (Å, º)

Cg1 and Cg2 are the centroids of the C9–C11/C11^i^/C12^i^/C13^i^ and C11–C13/C9^i^/C10^i^/C11^i^ rings, respectively [symmetry code: (i) -x, -y + 1, -z + 1]

**Table:**

*D*—H···*A*	*D*—H	H···*A*	*D*···*A*	*D*—H···*A*
N1—H1···O2^ii^	0.86	1.86	2.704 (3)	165
N2—H2···O1	0.86	1.88	2.684 (3)	155
C8—H8*E*···O3^iii^	0.96	2.32	3.230 (4)	158
C4—H4···*Cg*1^iv^	0.93	2.61	3.468 (3)	154
C4—H4···*Cg*2^v^	0.93	2.61	3.468 (3)	154

Symmetry codes: (ii) *x*, *y*, *z*−1; (iii) −*x*+1, −*y*+1, −*z*+1; (iv) −*x*, −*y*, −*z*; (v) *x*, *y*−1, *z*−1.
